# Velocity and acceleration freely tunable straight-line propagation light bullet

**DOI:** 10.1038/s41598-020-68478-1

**Published:** 2020-07-13

**Authors:** Zhaoyang Li, Junji Kawanaka

**Affiliations:** Institute of Laser Engineering, Osaka University, 2-6 Yamadaoka, Suita, Osaka 565-0871 Japan

**Keywords:** Optics and photonics, Optical physics

## Abstract

Three-dimensional (3-D) light solitons in space–time, referred to as light bullets, have many novel properties and wide applications. Here we theoretically show how the combination of diffraction-free beam and ultrashort pulse spatiotemporal-coupling enables the creation of a straight-line propagation light bullet with freely tunable velocity and acceleration. This light bullet could propagate with a constant superluminal or subluminal velocity, and it could also counter-propagate with a very fast superluminal velocity (e.g., − 35.6*c*). Apart from uniform motion, an acceleration or deceleration straight-line propagation light bullet with a tunable instantaneous acceleration could also be produced. The high controllability of the velocity and the acceleration of a straight-line propagation light bullet would enable very specific applications, such as velocity and/or acceleration matched micromanipulation, microscopy, particle acceleration, radiation generation, and so on.

## Introduction

The combination of diffraction-free beam and dispersion-free pulse permits the chance to produce 3-D self-similar spatiotemporal optical wave packets (light bullets), which could propagate over long distances and, importantly, maintain invariant intensity profiles in space and time^1–4^. In nonlinear optics, the balance between nonlinearity and dispersion/diffraction could easily result in temporal/spatial solitons^5–13^, however some applications require to generate light bullets in linear/free space. In linear optics, Bessel beam is one of the most important diffraction-free beams whose central core is propagation invariant^14,15^, and up to now many novel methods have already been proposed^16–21^, such as circular slit with lens^16^, axicon^17^, spatial light modulator^18^, and so on. Airy beam is another interesting diffraction-free beam, and, compared with Bessel beam, its main intensity maxima and lobes could tend to accelerate during propagation along a parabolic trajectory^22–29^. For a diffraction-free beam, when the monochromatic wave is replaced by a dispersion-free pulse, a light bullet might be produced. The simplest form is the Gauss-Bessel/Airy light bullet in free space (non-dispersion environment) or the Bessel/Airy-Bessel/Airy light bullet in materials (dispersion environment) ^1,22,30,31^. To control the velocity and even the acceleration of a light bullet is an interesting but challenging work. Previously, during a short propagation distance, the group velocity given by *υ*_*g*_ = *c*/*n*_g_ could be controlled by crafting the wavelength-dependent refractive index^32^, however some very special material or photonic systems are necessary^33–41^, such as ultra-cold atoms^33^, hot atomic vapors^35^, stimulated Brillouin scattering^36^, active gain resonances^37^, tunneling junctions^38^, metamaterials^39^, photonic crystals^40^, and so on. The problem is that, in transparent materials at wavelengths far from resonance or even in free space, the controllability of *n*_g_ and accordingly the group velocity *υ*_*g*_ would be very limited. Light bullet with an airy beam could self-accelerate during propagation in free space, however which travels along a bended (parabolic) propagation trajectory^22–24^. Recently, several spatiotemporal coupling methods have been proposed to control the group velocity of optical wave packets in free space^42–46^. For example by controlling temporal and spatial dispersion (temporal chirp and chromatic aberration)^42,44^, a flying focus with a tunable group velocity could be achieved, however the propagation distance is limited within the focusing region. In this article, we combined the first-order spatiotemporal coupling (pulse-front pre-deformation) with the diffraction-free (Gauss-Bessel) pulsed beam and theoretically produced a straight-line propagation light bullet in free space, whose velocity and acceleration could be freely controlled, including all cases of superluminal, subluminal, acceleration, deceleration and backwards-traveling group velocities. We have mainly discussed the case of a Gauss-Bessel pulsed beam in free space, and the method is also suitable to other types of pulsed beams, for example the Airy-Bessel pulsed beam in free space or materials. This velocity and acceleration tunable straight-line propagation light bullet has lots of specific applications from basic sciences to industry applications.

### Superluminal and subluminal light bullets

**Figure 1:**
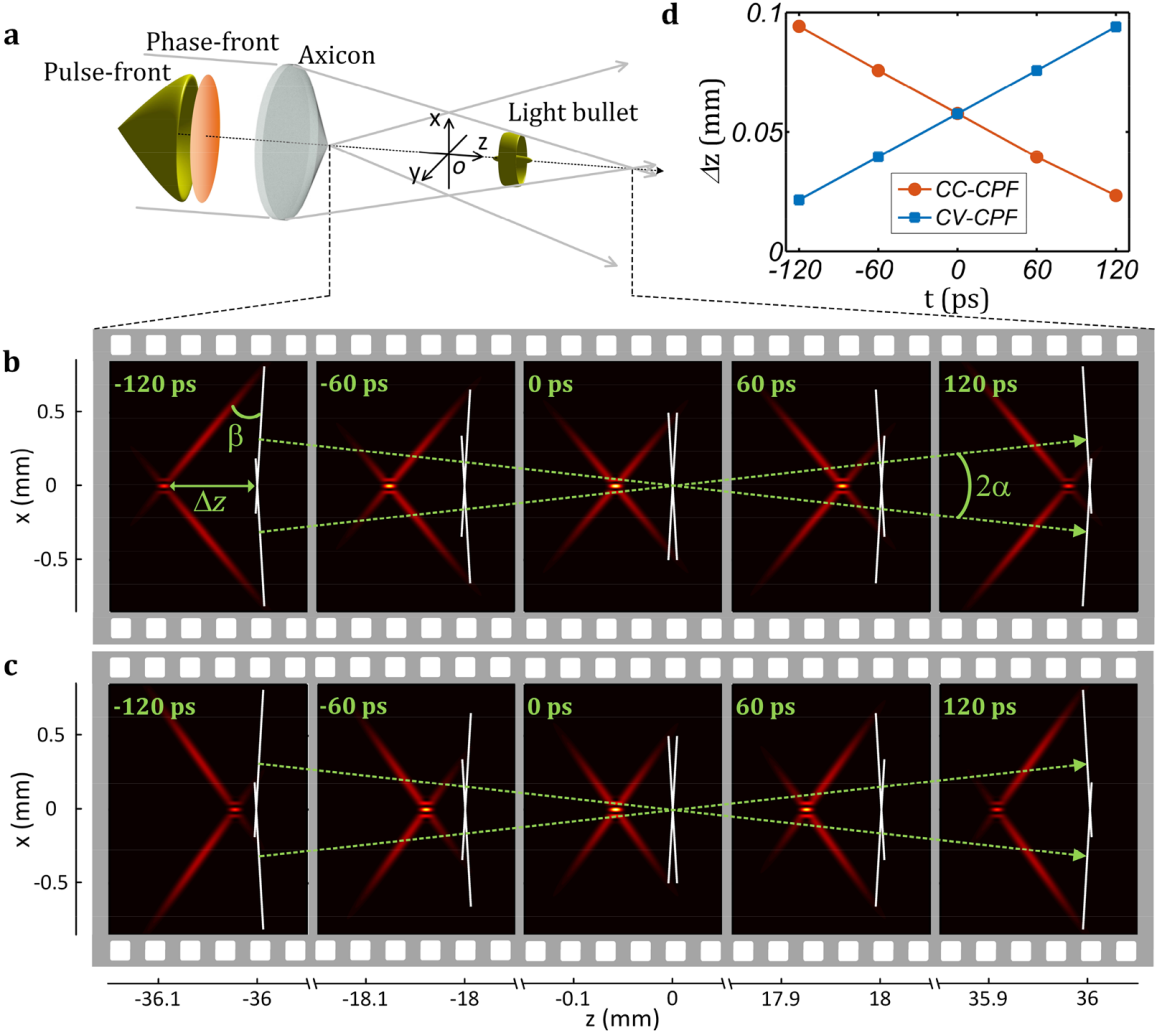
Gauss-Bessel light bullet with a conical-pulse-front pre-deformation. (**a**) The input pulsed beam possesses a plane phase-front and a conical pulse-front, and the axicon generates a Gauss-Bessel light bullet (in free space) in the interference region. (**b**) Superluminal light bullet in the case of CC-CPF (*α* = 0.5° and *β* = 6.6°). The longitudinal gap *Δ*z (between the light bullet and the intersection of phase-fronts illustrated by white lines) decreases during propagation. (**c**) Subluminal light bullet in the case of CV-CPF (*α* = 0.5° and *β* = − 6.6°). The longitudinal gap *Δ*z increases during propagation. (**d**) Variation of the longitudinal gap *Δ*z with time *t*. The travelling velocity of the intersection of phase-fronts is 1.00004*c*, and that of the light bullet with CC-CPF of (**b**) and CV-CPF of (**c**) is 1.001*c* and 0.999*c*, respectively.

An axicon can transform a plane wave into a conical wave and generate a Bessel beam in the overlap region due to interference^17^. In vacuum, the propagation velocity (group velocity) of this Bessel beam is *υ*_*b*_ = *c*/cos*α*, where *c* is the light speed in vacuum and *α* is the (half) conical angle (relevant to both the refractive index and the wedge angle of the axicon)^47^. It can be found that this velocity is faster than the light speed in vacuum *c*, which also increases with increasing the conical angle *α*. Figure 1a shows the key idea of the method proposed in this article that the input pulsed beam possesses a distortion-free (plane) phase-front but a pre-deformed (conical) pulse-front, which is quite different from the previous case with both distortion-free (plane) phase- and pulse-fronts. It is necessary to introduce that the phase-front is the surface perpendicular to the propagation direction while the pulse-front is the surface coinciding with the peak of a pulse, which are respectively determined by the phase- and group-velocities^48^. The result of the method is that the velocity of the generated light bullet could be freely controlled. As an example we begin the simulation with an initial input pulsed beam with a 30 fs (FWHM) pulse in time, a 2 mm (1/e^2^) beam in space and a concave conical-pulse-front (CC-CPF) in space–time, and the generation and the propagation of the light bullet in the 2-D propagation section of the *x–z* plane is shown in Fig. 1b. The axicon spatially divides the input pulsed beam into two Gauss-Gauss ones and changes their travelling directions (green arrows) with symmetrical angles of *α* =  ± 0.5°. The phase-fronts of two pulsed beams are illustrated by white lines, and the pulse-fronts are shown by red distributions. It can be found that in the overlap region a light bullet (in free space) is generated due to interference, which in time is a Gauss pulse and in space is a Bessel beam. Because the pulse-front is pre-deformed to deviate from the phase-front with a tilt angle of *β* = 6.6°, the generated light bullet is not located at the intersection of the phase-fronts anymore, and we can say the light bullet is temporally or spatially delayed along the longitudinal axis. Here, we make two definitions: in space, the propagation (longitudinal) axis of the Bessel beam is the *z*–axis and its geometrical center in the overlap region (formed by two pulsed beams after the axicon) is the origin of *z* = 0; and in time, the moment when the intersection of two phase-fronts (the original location of the light bullet if without any pulse-front pre-deformation) arriving at *z* = 0 is the zero time of *t* = 0. Figure 1b gives the detailed distributions of the pulse-fronts, the phase-fronts and the light bullet at different times of *t* = − 120, − 60, 0, 60 and 120 ps during propagation. According to the previous result^41^, the intersection of two phase-fronts travels at a velocity of 1.00004*c* governed by *υ*_*b*_ = *c*/cos*α*. However, due to the pulse-front pre-deformation, the light bullet is behind the intersection of phase-fronts and the longitudinal gap *Δ*z (distance between the light bullet and the intersection of phase-fronts) decreases with time during propagation. This phenomenon indicates the travelling velocity of the light bullet is faster than that of the intersection of phase-fronts, i.e., a superluminal light bullet is produced. Red curve in Fig. 1d shows the variation of the longitudinal gap *Δ*z with time *t* is linear, and accordingly the light bullet should possess a constant velocity. The simulated value is 1.001*c* which is faster than the velocity of the intersection of phase-fronts of 1.00004*c*. In geometrical optics, within the Rayleigh range, the velocity of this light bullet satisfies

1 $$ \upsilon_{b} = c\frac{\cos \beta }{{\cos \left( {\alpha + \beta } \right)}}, $$

which is governed by both the conical angle *α* (determined by the axicon) and the pulse-front tilt angle *β* (determined by the pulse-front pre-deformation). Thus, the pulse-front tilt angle *β* is another degree of freedom to control the velocity *υ*_*b*_ of the light bullet.

In another case, when a convex conical-pulse-front (CV-CPF) with *β* = − 6.6° is pre-deformed and all the other parameters remain unchanged, Fig. 1c shows although the light bullet is still behind the intersection of the phase-fronts, the longitudinal gap *Δz* is gradually increasing (instead of decreasing in Fig. 1b) during propagation. Therefore, the velocity of this light bullet is slower than that of the intersection of phase-fronts. The detailed variation of the longitudinal gap *Δz* with time *t* is shown by blue curve in Fig. 1d, which is linear, too (constant velocity). The simulated value is 0.999*c* and slower than the velocity of the intersection of phase-fronts of 1.00004*c*, and accordingly a subluminal light bullet is produced.

In applications, the above superluminal and subluminal velocities for the cases of CC-CPF and CV-CPF could be calculated by using Eq. (1) with a positive and a negative pulse-front tilt angle *β*, respectively, and thereby Eq. (1) can be directly used to describe the light bullet created by using this method.

**Figure 2:**
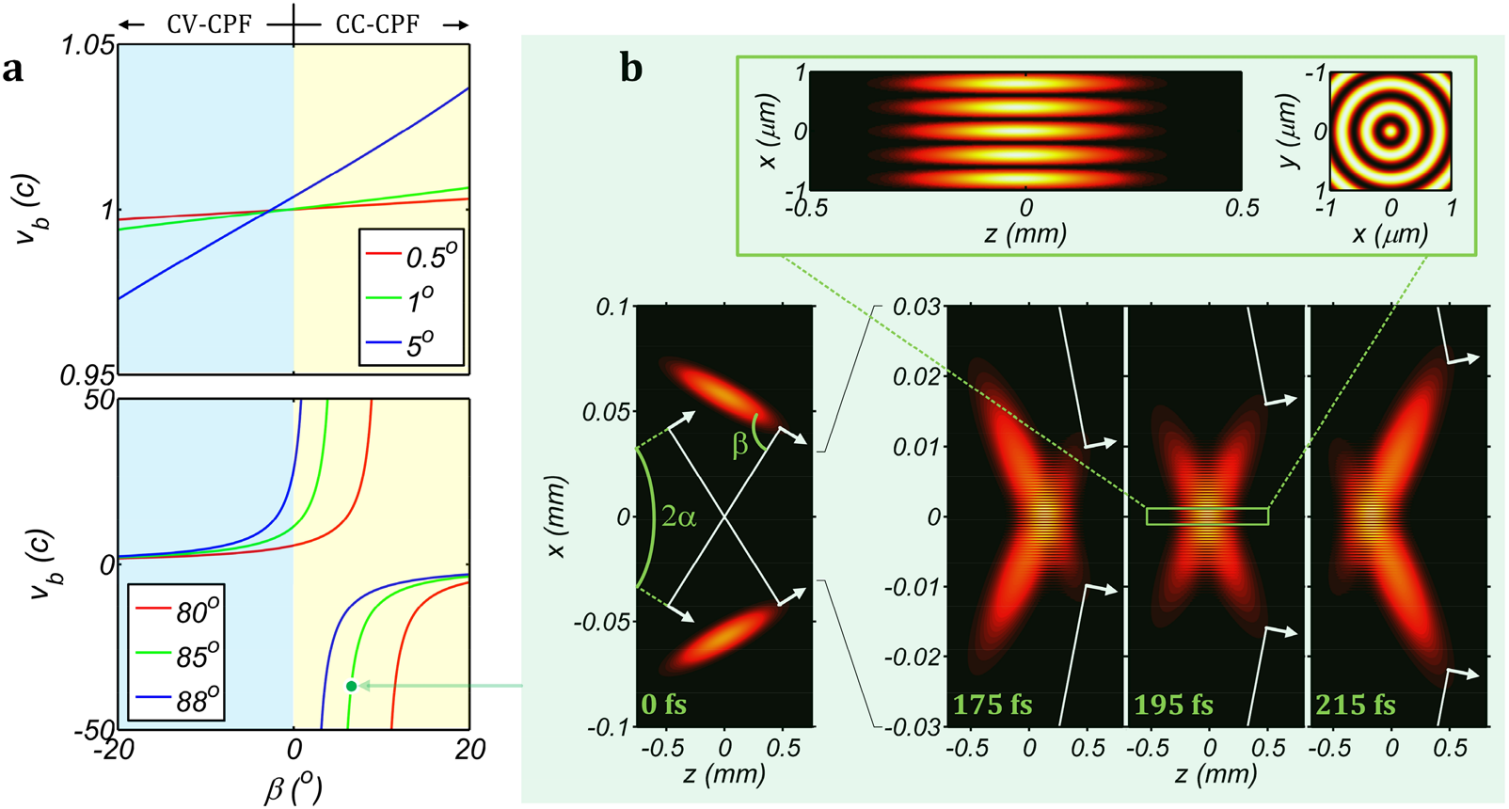
Controllability of velocity and backwards travelling light bullet. (**a**) Velocity of the light bullet as a function of the pulse-front tilt angle *β* for different conical angles *α* (*α* equals 0.5°, 1° & 5° in upper and 80°, 85° & 88° in lower). The velocity is normalized by *c*. (**b**) Backwards travelling light bullet with *α* = 85° and *β* = 6.6° (spot marked in (**a**) lower), and the velocity is − 35.6*c*. Due to a large conical angle *α*, the light bullet is very small and most energies are transferred to the rings around the light bullet. White lines and arrows in (**b**) illustrate phase-fronts and propagation directions.

Next, we continue to the controllability of the light bullet velocity. Figure 2a shows the variation of the light bullet velocity *υ*_*b*_ with the pulse-front tilt angle *β* for different conical angles *α*. When the conical angle *α* is small (for example 0.5°, 1° and 5° in Fig. 2a upper), the light bullet velocity *υ*_*b*_ and the pulse-front tilt angle *β* satisfy a linear relationship, and a superluminal or a subluminal light bullet could be produced by choosing a positive (CC-CPF) or a negative (CV-CPF) pulse-front tilt angle *β*. The sensitivity of the light bullet velocity *υ*_*b*_ to the pulse-front tilt angle *β* could be increased by increasing the conical angle *α*. Once the conical angle *α* is dramatically increased (for example 80°, 85° and 88° in Fig. 2a lower), the linear relationship disappears, and the light bullet velocity *υ*_*b*_ becomes very sensitive to the pulse-front tilt angle *β*, especially when *α* + *β* is close to 90°. And even a negative velocity *υ*_*b*_ (backwards travelling) could be generated, when *α* + *β* is large than 90°. We simulate this case, when the conical angle *α* is increased to 85° and all the other parameters used in Fig. 1b remain unchanged (the spot marked in Fig. 2a lower). Figure 2b shows the generation and the propagation of the light bullet in the *x–z* plane. At *t* = 0 two phase-fronts intersect at the position of *z* = 0, while two pulse-fronts still separate in space. From *t* = 175 fs to 195 fs and then to 215 fs, two pulse-fronts begin to intersect with each other at the leading edge and then the intersection quickly moves towards the trailing edge, showing a backwards travelling light bullet in space–time. The propagation velocity of this light bullet is very high of − 35.6*c*. This phenomenon could be explained by Eq. (1). Because the sum of the conical angle and the pulse-front tilt angle *α* + *β* is slightly larger than 90° (91.6°) and the pulse-front tilt angle *β* is small (6.6°), so the light bullet velocity *υ*_*b*_ is negative and its absolute value is dramatically increased. The problem of this backwards travelling light bullet is that, once a large conical angle *α* is used, the size of the center lobe of the Bessel beam (the light bullet) would be greatly reduced and most energies would transfer from the center to side lobes (concentric rings around the light bullet in Fig. 2b)^15^. In some applications, such as laser drilling, optical tweezing, etc., the energy loss at the center lobe might be unacceptable, however in other applications, such as particle manipulation, secondary-radiation generation, etc., a superluminal backwards travelling light bullet, as well as a series of concentric rings, may possess unique performance.

### Acceleration and deceleration light bullets

**Figure 3:**
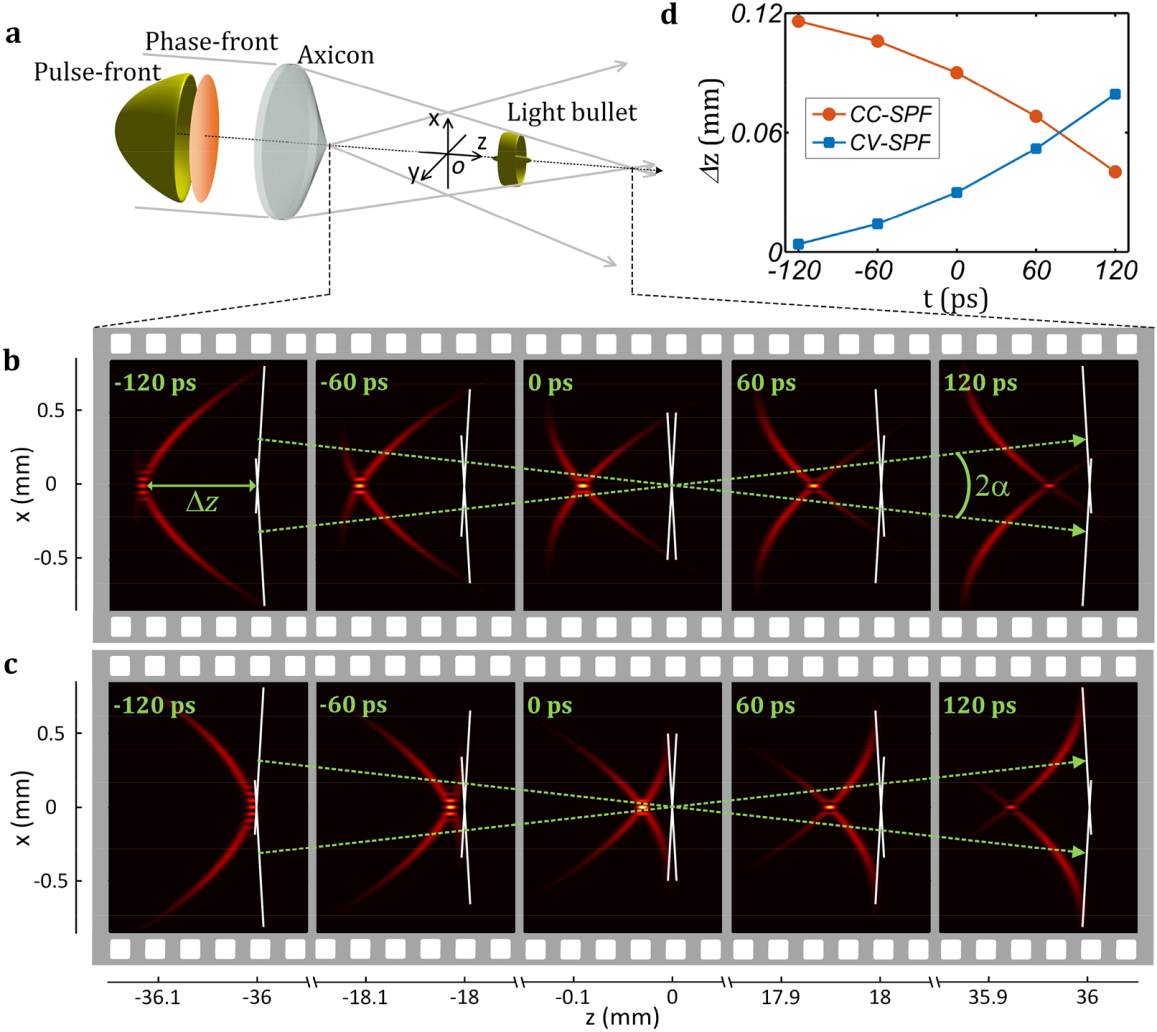
Gauss-Bessel light bullet with a spherical-pulse-front pre-deformation. (**a**) The input pulsed beam possesses a plane phase-front and a spherical pulse-front, and the axicon generates a Gauss-Bessel light bullet (in free space) in the interference region. (**b**) Acceleration light bullet in the case of CC-SPF (*α* = 0.5° and *R* = − 4.2 mm). *R* is the spherical-pulse-front curvature. The longitudinal gap *Δ*z (between the light bullet and the intersection of phase-fronts illustrated by white lines) decreases faster and faster during propagation. (**c**) Deceleration light bullet in the case of CV-SPF (*α* = 0.5° and *R* = 4.2 mm). The longitudinal gap *Δ*z increases faster and faster during propagation. (**d**) Variation of the longitudinal gap *Δ*z with time *t*. The travelling velocity of the intersection of phase-fronts is 1.00004*c*, and the nonlinear decreasing and increasing of the longitudinal gap *Δ*z illustrates acceleration and deceleration for the case of CC-SPF of (**b**) and CV-SPF of (**c**) respectively.

When the initial input pulsed beam possesses a spherical-pulse-front and a plane-phase-front (Fig. 3a), which could be frequently found in a transmission telescope and usually called as “pulse-front-curvature”^48,49^ or “radial-group-delay”^50^, the velocity of the generated light bullet would not be a constant during propagation. The simulation is still based on the parameters used in the above section. First we simulate the case of concave spherical-pulse-front (CC-SPF), and the curvature *R* is − 4.2 mm. Figure 3b shows the distributions of the pulse-fronts, the phase-fronts and the generated light bullet at different times *t* in the *x–z* plane. The light bullet is still behind the intersection of the phase-fronts, the longitudinal gap *Δ*z also decreases during propagation (superluminal light bullet), however the decreasing amount in the first half propagation (from *t* = − 120 ps to 0) is very small while in the second half propagation (from *t* = 0 to 120 ps) becomes much obvious. Because the intersection of the phase-fronts has a constant velocity of 1.00004*c*, then the light bullet experiences an acceleration propagation. Red curve in Fig. 3d gives the detailed variation of the longitudinal gap *Δ*z with time *t*, which is no longer a perfect linear relationship anymore. The longitudinal gap *Δ*z decreases faster and faster with time *t*, and consequently an acceleration light bullet is produced. Second we simulate another case of convex spherical-pulse-front (CV-SPF) with a curvature *R* of 4.2 mm. Figure 3c shows the generation and the propagation of the corresponding light bullet, and the longitudinal gap *Δ*z is increasing during propagation, which also becomes more and more obvious with the propagation position *z* or time *t*. Blue curve in Fig. 3d shows the variation of the longitudinal gap *Δ*z with time *t* and illustrates that the longitudinal gap *Δ*z increases faster and faster with time *t*, and therefore a deceleration light bullet is produced.

The acceleration or the deceleration propagation of a light bullet could be explained by Eq. (1). The spherical-pulse-front can be considered as the superposition of a series of conical-pulse-fronts. Figures 3b,c show that, during propagation, different parts of two pulse-fronts contribute to the creation of the light bullet, which corresponds to different pulse-front tilt angles *β* at different propagation times *t* or positions *z*, and consequently, the velocity *υ*_*b*_ varies with time *t* or position *z* during propagation, showing acceleration or deceleration.

**Figure 4:**
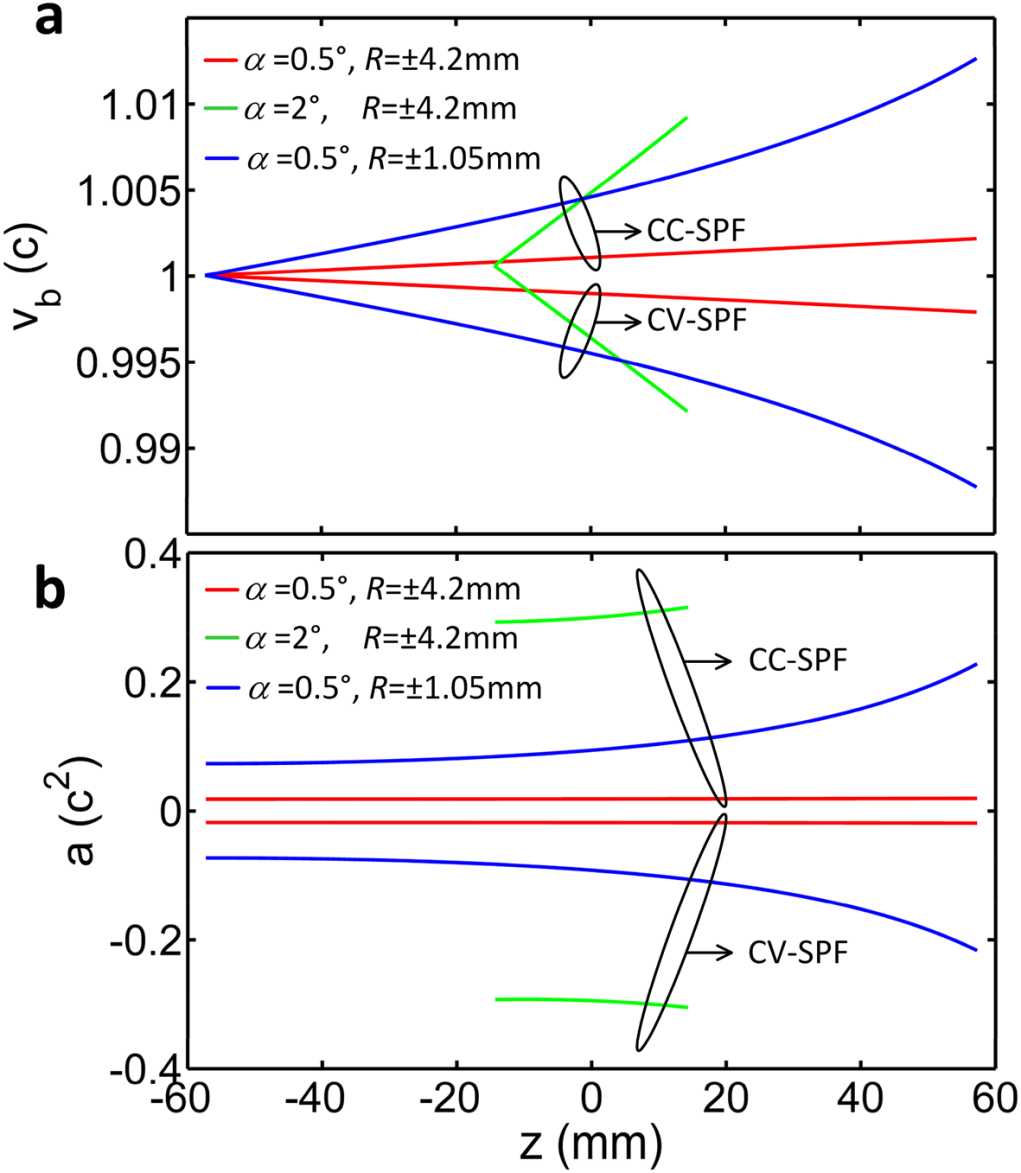
Controllability of velocity and acceleration. (**a**,**b**) Propagation position *z* dependent velocity *υ*_*b*_ and acceleration *a* of a light bullet when the conical angle *α* is increased from 0.5° to 2° and the spherical-pulse-front curvature *R* is changed from ± 4.2 mm to ± 1.05 mm (positive and negative *R* for CV-SPF and CC-SPF, respectively), respectively. The velocity *υ*_*b*_ and the acceleration *a* is normalized by *c* and *c*^2^, respectively.

The propagation position *z* (or time *t*) dependent velocity *υ*_*b*_ and acceleration *a* of a light bullet for different geometries are simulated based on the parameters used in above, and the result is shown in Fig. 4. When the conical angle *α* is 0.5° and the spherical-pulse-front curvature *R* is ± 4.2 mm (negative and positive for CC-SPF and CV-SPF, respectively), red curves in Fig. 4a,b illustrate the variation of the velocity *υ*_*b*_ and the acceleration *a* of CC-SPF (acceleration) and CV-SPF (deceleration) with the propagation position *z*. To analyze the influence of the conical angle *α* and the curvature *R*, we modify the two parameters individually. When the conical angle *α* is enlarged by four times from 0.5° to 2°, green curves show that: first the tunable range of the velocity *υ*_*b*_ is increased, and second the absolute value of the acceleration *a* is enlarged (significant acceleration or deceleration). However, the tunable range of the acceleration *a* during propagation is not increased, but unfortunately the propagation distance of the light bullet (Bessel beam) is reduced a lot which is determined by the conical angle *α*. When the absolute value of the spherical-pulse-front curvature *R* is reduced by four times from 4.2 mm to 1.05 mm, blue curves illustrate that: first the tunable ranges of both the velocity *υ*_*b*_ and the acceleration *a* are dramatically increased, and more importantly the long propagation distance of the light bullet (Bessel beam) remains unchanged. In this case, adjusting the spherical-pulse-front curvature *R* is an ideal approach to control the propagation position (or time) dependent velocity *υ*_*b*_ and acceleration *a* of an acceleration or deceleration light bullet, which would be very attractive in some special applications, such as particle acceleration^51,52^, plasma channel generation^53^, and so on.

## Discussion and conclusion

In this article, we introduced the method based on a Gauss-Bessel pulsed beam, which in free space can be considered as diffraction-free and dispersion-free (light bullet). According to previous publications^1^, if the third-order dispersion (cubic spectral phase) is introduced into the initial pulse, the Gauss-Bessel pulsed beam would be conveniently transferred into an Airy-Bessel pulsed beam, which is a light bullet in dispersion materials. This process won’t influence the method and the result proposed here, and consequently a velocity and acceleration tunable straight-line propagation light bullet in dispersion materials could also be produced. In principle, this method is applicable to any other type of pulsed beams, and we won’t repeat the detail again.

In conclusion, we have theoretically proposed a method that by combining the traditional diffraction-free beam (Bessel beam in this article) with the first-order spatiotemporal coupling ultra-shot pulse (pulse-front pre-deformation), a straight-line propagation light bullet with freely tunable velocity (superluminal and subluminal) and acceleration (acceleration and deceleration) could be produced, and a backwards traveling superluminal light bullet could also be created. This highly tunable light bullet has a broad range of applications, for example a superluminal or subluminal light bullet is quite useful to time-dependent pump-probe measurement/microscopy, and an acceleration or deceleration light bullet can be used to match a flying particle in particle acceleration experiment.

## Methods

### Pulse-front pre-deformation

**Figure 5:**
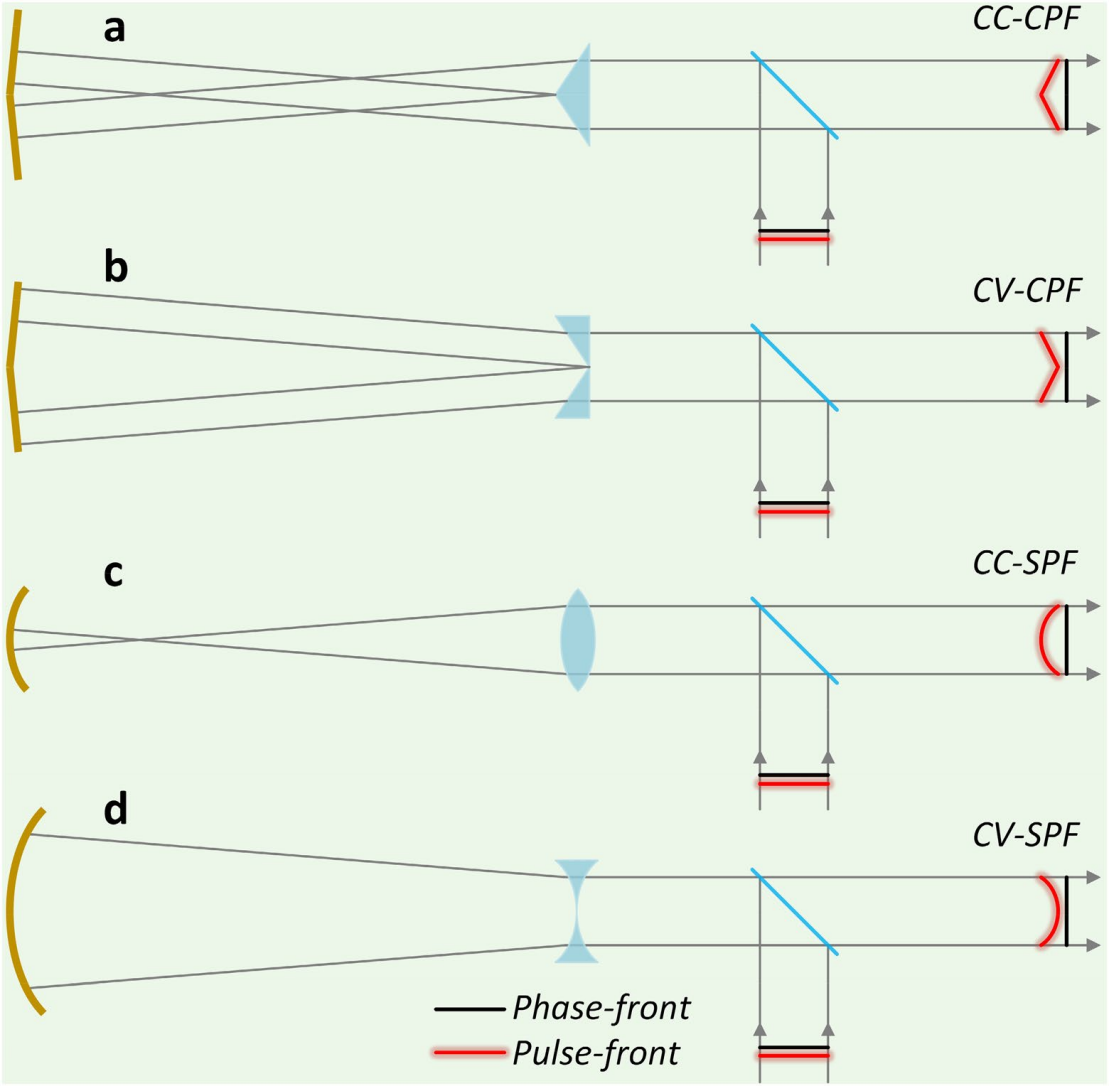
Pulse-front pre-deformation. The combination of transmission and reflection optics could be used to generate (**a**) concave conical-pulse-front (CC-CPF), (**b**) convex conical-pulse-front (CV-CPF), (**c**) concave spherical-pulse-front (CC-SPF), and (**d**) convex spherical-pulse-front (CV-SPF), while the plane phase-front remains unchanged.

The pulse-front of the initial input pulsed beam can be easily deformed by using a pair of matched transmission and reflection optics^48–50,54–56^, and its phase-front would remain unchanged in this process due to the perfect imaging geometry. Figure 5a shows a pair of transmission convex axicon and concave conical reflector would generate CC-CPF, and similarly CV-CPF can be produced by a pair of transmission concave axicon and concave conical reflector (Fig. 5b). When the transmission convex or concave axicon and the concave conical reflector is respectively replaced by a transmission convex or concave spherical lens and a concave spherical reflector, CC-SPF (Fig. 5c) or CV-SPF (Fig. 5d) would be produced.

### Coordinate rotation

The axicon transfers an input plane wave into an output conical wave, and, in the 2-D propagation section, the upper and lower half beams have their individual travelling directions, which are symmetrical bout the Bessel beam propagation axis (Figs. 1a or 3a). The input plane wave and the generated Bessel beam are described in the coordinate system of *r-z*, where *r* is the radial axis and *z* is the propagation axis. The output upper or lower half beam after the axicon is described in its own propagation coordinate system of *r*_*α*_-*z*_*α*_, where *r*_*α*_ is the radial axis and *z*_*α*_ is the propagation axis. The origins of two coordinate systems have a same location, which is at the geometrical center of the overlap region after the axicon. Thus, two coordinate systems of *r-z* and *r*_*α*_-*z*_*α*_ satisfy the rotation relationship

2 $$ \left[ {\begin{array}{*{20}c} {r_{\alpha } } \\ {z_{\alpha } } \\ \end{array} } \right] = \left[ {\begin{array}{*{20}c} {\cos \alpha } & { - \sin \alpha } \\ {\sin \alpha } & {\cos \alpha } \\ \end{array} } \right]^{ - 1} \left[ {\begin{array}{*{20}c} r \\ z \\ \end{array} } \right], $$

where *α* is the half conical angle which is also the rotation angle of the coordinate system of *r*_*α*_-*z*_*α*_. In this article, positive and negative coordinate rotation angles *α* are defined for the upper and lower half beams after the axicon in the 2-D propagation section, respectively.

### Pulsed beam propagation and coherent superposition

After the axicon, the *E*-field of upper or lower half beam in the 2-D propagation section of *r*_*α*_*-z*_*α*_ can be described as

3 $$ E\left( {r_{\alpha } ,z_{\alpha } ,\tau } \right) = A\exp \left( { - \frac{{\left( {\tau - T\left( {r_{\alpha } } \right) - t} \right)^{2} }}{{\Delta \tau^{2} }}} \right)\exp \left( { - ik\frac{{r_{\alpha }^{2} }}{{2q_{{z_{\alpha } }} }} - ikz_{\alpha } + i\phi } \right), $$

where *A* is the amplitude, *τ* is the local time, *T*(*r*_*α*_) is the spatiotemporal coupling term, *t* and *z*_*α*_ (*t* = *z*_*α*_/*c*) are time and length from the *z*_*α*_–axis origin of *z*_*α*_ = 0 to the current propagation position, *Δτ* is the pulse duration, *k* is the wave vector, *q*_*zα*_ is the complex Gaussian beam parameter at *z*_*α*_, and *ϕ* is the initial phase. In this article, the beam waist locates at the coordinate origin of (*r*_*α*_ = 0*, z*_*α*_ = 0).

For the case of conical pulse-front, the spatiotemporal coupling term in Eq. (3) is given by

4 $$ T\left( {r_{\alpha } } \right) = \left\{ {\begin{array}{*{20}l} {\frac{\tan \beta }{c}\left( {r_{\alpha } \mp w_{{z_{\alpha } }} } \right)} \hfill &\quad {CC - CPF} \hfill \\ {\frac{\tan \beta }{c}\left( {r_{\alpha } \pm w_{{z_{\alpha } }} } \right)} \hfill &\quad {CV - CPF} \hfill \\ \end{array} } \right., $$

where *w*_*zα*_ is the beam waist, and we should emphasize that it is the beam waist of the upper or lower half beam after the axicon. For the case of CC-CPF, the upper or lower half beam after the axicon has a positive and negative pulse-front tilt angle *β*, respectively, which corresponds to -*w*_*zα*_ and + *w*_*zα*_. While for the case of CV-CPF, the situation is exactly the opposite: the pulse-front tilt angle *β* of the upper and lower half beam is negative and positive, respectively, and which corresponds to + *w*_*zα*_ and -*w*_*zα*_.

For the case of spherical pulse-front, the spatiotemporal coupling term in Eq. (3) satisfies

5 $$ T\left( {r_{\alpha } } \right) = \left\{ {\begin{array}{*{20}l} {\frac{{4w_{{z_{\alpha } }}^{2} - \left( {r_{\alpha } \pm w_{{z_{\alpha } }} } \right)^{2} }}{2Rc}} \hfill &\quad {CC - SPF} \hfill \\ { - \frac{{\left( {r_{\alpha } \pm w_{{z_{\alpha } }} } \right)^{2} }}{2Rc}} \hfill &\quad {CV - SPF} \hfill \\ \end{array} } \right., $$

where *R* is the curvature of the pulse-front, which is negative and positive for CC-SPF and CV-SPF, respectively. And + *w*_*zα*_ and -*w*_*zα*_ correspond to the upper and lower half beams after the axicon.

The complex Gaussian beam parameter *q*_*zα*_ at *z*_*α*_, containing the information of both the radius of beam curvature *R*_*zα*_ and the beam size *w*_*zα*_, satisfies

6 $$ \frac{1}{{q_{{z_{\alpha } }} }} = \frac{1}{{R_{{z_{\alpha } }} }} - i\frac{2}{{kw_{{z_{\alpha } }}^{2} }}. $$

After propagation governed by Eq. (3) in the *r*_*α*_-*z*_*α*_ coordinate system, the coherently superimposed (interference) *E*-field in the *r*-*z* coordinate system is given by

7 $$ E_{s} \left( {r,z,\tau } \right) = E_{u} \left( {r,z,\tau } \right) + E_{l} \left( {r,z,\tau } \right), $$

where *E*_u_ and *E*_l_ are *E*-fields of two half beams after the axicon.

## Data Availability

The data that support the findings of this study are available from the corresponding author upon reasonable request.
